# Clinical implication of monitoring regional ventilation using electrical impedance tomography

**DOI:** 10.1186/s40560-019-0358-4

**Published:** 2019-01-18

**Authors:** Atsuko Shono, Toru Kotani

**Affiliations:** 1 Department of Anesthesiology, Shimane University, 89-1 Enya-cho, Izumo City, Shimane 693-8501 Japan; 2 Department of Intensive Care Medicine, Showa University School of Medicine, 1-5-8 Hatanodai, Shinagawa-ku, Tokyo, 142-8666 Japan

**Keywords:** Electrical impedance tomography, Regional ventilation monitoring, Ventilator-associated lung injury

## Abstract

Mechanical ventilation can initiate ventilator-associated lung injury (VALI) and contribute to the development of multiple organ dysfunction. Although a lung protective strategy limiting both tidal volume and plateau pressure reduces VALI, uneven intrapulmonary gas distribution is still capable of increasing regional stress and strain, especially in non-homogeneous lungs, such as during acute respiratory distress syndrome. Real-time monitoring of regional ventilation may prevent inhomogeneous ventilation, leading to a reduction in VALI.

Electrical impedance tomography (EIT) is a technique performed at the patient’s bedside. It is noninvasive and radiation-free and provides dynamic tidal images of gas distribution. Studies have reported that EIT provides useful information both in animal and clinical studies during mechanical ventilation. EIT has been shown to be useful during lung recruitment, titration of positive end-expiratory pressure, lung volume estimation, and evaluation of homogeneity of gas distribution in a single EIT measure or in combination with multiple EIT measures. EIT-guided mechanical ventilation preserved the alveolar architecture and maintained oxygenation and lung mechanics better than low-tidal volume ventilation in animal models. However, careful assessment is required for data analysis owing to the limited understanding of the results of EIT interpretation. Previous studies indicate monitoring regional ventilation by EIT is feasible in the intensive care setting and has potential to lead to lung protective ventilation. Further clinical studies are warranted to evaluate whether monitoring of regional ventilation using EIT can shorten the duration of ventilation or improve mortality in patients with acute respiratory distress syndrome.

## Introduction

Acute respiratory distress syndrome (ARDS) is not rare in the intensive care unit and has been studied for decades; however, ARDS-related mortality remains unacceptably high. Mechanical ventilation (MV) is often required to provide adequate gas exchange in patients with ARDS but can injure the lungs and cause poor outcomes [1]. Mechanical stress alters lung physiology and induces local inflammatory responses, leading to initiate ventilator-associated lung injury (VALI) that causes multiple organ dysfunction through the production of inflammatory mediators. VALI plays a pivotal role in developing multiple system organ failure (MSOF) in ARDS, indicating that minimizing VALI is a key to reducing mortality during ARDS.

Alveolar overdistension and/or repetitive collapse and reopening of alveoli are responsible for the development of VALI. The use of low tidal volumes and limiting plateau pressures, referred to as lower tidal volume ventilation (LTV) in association with permissive hypercapnia [2], decreased inflammatory cytokine production, increased organ failure free days, and improved mortality of patients with ARDS [3]. As LTV is feasible and requires neither special ventilators nor specially trained staff, it is considered essential for the management of ARDS. However, a prospective clinical study [4] showed tidal hyperinflation occurred in one third of patients with acute lung injury (ALI) even though they were ventilated with LTV. In the study, inflammatory cytokines in the airway significantly increased in hyperinflated lungs. They also showed that concurrent inflation and deflation of a tidal breath during an inspiration could occur at different sites in a ventilated lung with a large nonaerated area. Although the authors did not refer, the finding clearly indicates that uneven intrapulmonary gas distribution during LTV still can increase regional stress and strain, especially in non-homogeneous lungs, as with ARDS. It is known, in general, that the possible presence of stress raisers and lung inhomogeneity during tidal ventilation increases dynamic lung strain [5]. In an animal model, it has been shown that the increased dynamic strain was more associated with the development of pulmonary edema, derangement of lung mechanics, and higher mortality than static strain [6]. To detect uneven distribution of ventilation increasing strain, monitoring of regional ventilation is crucial. Previous studies using computed tomography have shown the importance of regional ventilation monitoring [4, 7]. However, clinical use is limited because of radiation exposure and the risk associated with patient transportation. Furthermore, computed tomography provides static information, making it difficult to analyze the time delay of tidal inflation.

Electrical impedance tomography (EIT) is a clinically available, noninvasive technique that provides dynamic tidal images of gas distribution at the patient’s bedside [8]. EIT enables frequent adjustment of ventilator settings because of its radiation-free nature and dynamic assessment. Studies have reported that EIT provides useful information, such as lung recruitment, positive end-expiratory pressure (PEEP) adjustment, lung volume estimation, and homogeneity of gas distribution during MV. It has been available commercially in Europe since 2011, but the Pharmaceuticals and Medical Devices Agency in Japan has not approved its clinical use at this moment. Therefore, in Japan, clinical research has just begun a few years ago and large-volume research has not been conducted yet.

In this narrative review, the authors summarize the animal and clinical data to assess the feasibility and efficacy of monitoring regional ventilation by EIT. Additionally, the roles of EIT to improve lung protective ventilation management for patients with and at risk of ARDS are discussed.

## Visualization of ventilation distribution by EIT

EIT is a monitoring tool that can visualize the ventilation distribution by measuring the regional increase in impedance caused by inspired gas [9, 10]. Biological tissue consists of specific compositions (e.g., lipids, water, electrolytes) with distinctive responses to an externally applied alternating electric current that are generally described as “bioimpedance” [9]. Air in the thorax acts as electrical resistors and increases regional impedance accompanied with the respiratory cycle [10]. EIT monitors these impedance changes in real time. The change in impedance is actually measured by applying a small alternating current through electrodes implemented in the EIT belt, which is normally placed at 5th to 6th intercostal level of the patient’s chest. The raw impedance data are profiled using an algorithm as the cross-sectional image of the lung, similar to computed  tomography. The constructed sequential images are projected on the screen as a real-time moving image that shows ventilation distribution allowing easy understanding of how the lungs are ventilated. The EIT user recognizes the spatial localization and temporal discordance or asynchrony of ventilation by watching the screen at the patient’s bedside. To make the EIT image functional, an appropriate evaluation of the degree of heterogeneity of ventilation is required. Subsequent analysis using recorded impedance data enables the quantification of the spatial extent of regional distribution, its overall dispersion, and time delay as clinically distinctive parameters. Those values, calculated from the functional EIT image, are called EIT measures.

## EIT measures used in the literature

EIT measures to quantify the degree of inhomogeneous ventilation have been proposed. Several EIT measures adopt the same concept but are represented by using different computation methods and notations. A taxonomy of EIT terms and alternative terms with definitions and explanations has been provided in a previous review published in 2016 [8]. In the present review, EIT measures that have been frequently described and are well-documented in previous reports are divided into two groups: one consisting of spatial elements and the other including temporal elements. The following section describes each EIT measure.

### EIT measures for analyzing spatial distribution of ventilation

#### Tidal impedance variation (TIV)

Tidal impedance variation (TIV) represents impedance change generated by inspired gas during a tidal breath, calculated as the difference of impedance between the maximum and minimum values at end-inspiration and end-expiration.

$$ \mathrm{TIV}={\mathrm{Impedance}}_{\mathrm{max}}-{\mathrm{Impedance}}_{\mathrm{min}} $$

**Fig. 1:**
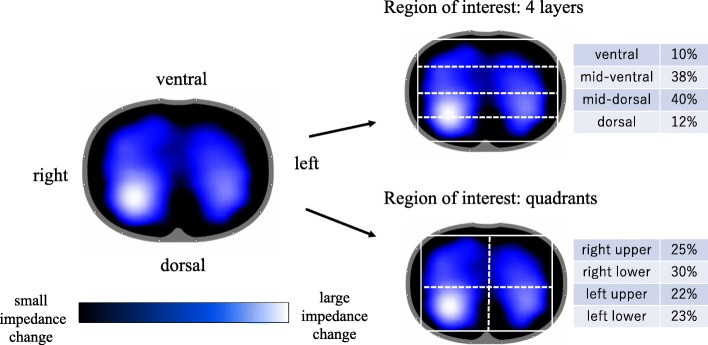
EIT tidal image*. Left,* typical EIT tidal image is shown. Regional distribution of tidal breath is visualized with a color scale based on calculated impedance changes during one breath. Brighter color (corresponding to large impedance change) shows a well-ventilated area. Darker color (small impedance change) shows a less ventilated area. *Right,* the images when region of interests are applied with horizontal layers (upper image) and quadrants (lower image). The distribution at each region (regional TIV) is expressed as a percentage of global tidal impedance variation (TIV)

Global TIV is the sum of impedance changes in all pixels across the whole image, which consists of a matrix of 32 × 32 pixels. It has been demonstrated that global TIV correlates with tidal volume. Regional TIV, characterizing regional ventilation, is calculated by the sum of impedance changes in the pixels within the selected region of interest (ROI) in horizontal layers or quadrants (Fig. 1). TIV is a basic parameter from which various EIT measures are derived in the process of subsequent analysis.

#### Regional respiratory system compliance

During mechanical ventilation, compliance can be calculated using driving pressure and tidal volume. Though following the trend in compliance is crucial in managing respiratory failure where the lung mechanics change frequently, the information derived from only global parameters might be insufficient to recognize the physiologic phenomenon accurately. EIT can monitor local impedance changes in various regions sectionalized at the discretion of the user. Locally distributed volume in a certain lung region can be estimated by regional TIV, which makes it possible to assess the regional characteristic of a ventilated lung [11]. Regional compliance, one of the EIT measures, is calculated by dividing regional TIV by driving pressure monitored in parallel as below [12–15].

$$ {\mathrm{Compliance}}_{\mathrm{region}}=\frac{{\mathrm{TIV}}_{\mathrm{region}}}{\mathrm{Pressure}\ \mathrm{above}\ \mathrm{PEEP}} $$

By monitoring regional compliance in different lung areas, e.g., non-dependent versus dependent areas in the lung, one can realize how inhomogeneous ventilation is under certain ventilatory settings owing to non-uniform regional compliance.

Zick and colleagues reported that after induction of ALI in pigs, regional compliance of the lung area decreased despite PEEP being increased from 5 to 10 cmH_2_O [11]. Applying recruitment maneuvers (RM) and using higher PEEP improved compliance in ventral regions but not in dorsal regions, suggesting a more aggressive PEEP setting was required to prevent the lung collapse in the dorsal regions. Thus, inhomogeneous ventilation not only in ARDS lungs but also in various situations under mechanical ventilation has been assessed by regional compliance.

#### Overdistension and atelectasis/collapse (ODCL)

By monitoring regional compliance at the pixel level during a PEEP trial, one can assess the regional lung area that might have overdistension and/or collapse (ODCL). During a decremental PEEP trial with a fixed driving pressure, the compliance in each pixel changes in response to the level of PEEP. This shows the highest compliance and identifies the best pixel compliance to the specific level of PEEP. When regional (pixel) compliance reduced by lowering PEEP from a specific level, the behavior of its pixel compliance represents collapse; whereas, when a similar reduction of compliance is seen by increasing PEEP, that indicates overdistension. The degree of collapse and overdistension at each PEEP level is calculated according to the formulas. For the calculation of collapse, the change in compliance for each pixel at a given PEEP level compared with its best compliance across all PEEP steps is calculated first [15],

$$ {\mathrm{Collpase}}_{\mathrm{pixel}}\ \left(\%\right)=\frac{\left({\mathrm{Best}\ \mathrm{Compliacne}}_{\mathrm{pixel}}-{\mathrm{Current}\ \mathrm{Compliacne}}_{\mathrm{pixel}}\right)\times 100}{{\mathrm{Best}\ \mathrm{Compliance}}_{\mathrm{pixel}}} $$

Thereafter, the accumulated collapse for the entire lung at each PEEP step is calculated as a weighted average summed up for all collapsed pixels, where the weighting factor is the best pixel compliance estimated by applying the formula [15]:

$$ \mathrm{Cumulated}\ \mathrm{Collpase}\ \left(\%\right)=\frac{\sum_{\mathrm{i}=1}^{\mathrm{valid}\ \mathrm{pixels}}\left({\mathrm{Collapse}}_{\mathrm{pixel}}\left(\%\right)\times {\mathrm{Best}\ \mathrm{Compliacne}}_{\mathrm{pixel}}\right)}{\sum_{\mathrm{i}=1}^{\mathrm{valid}\ \mathrm{pixels}}{\mathrm{Best}\ \mathrm{Compliacne}}_{\mathrm{pixel}}} $$

(valid pixels, numbers of pixels included for analysis)

**Fig. 2:**
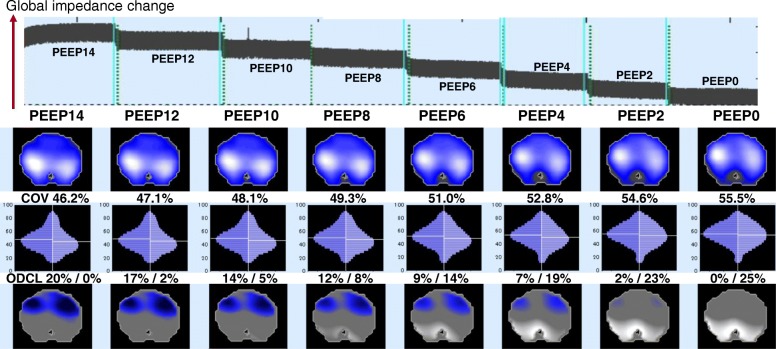
Decremental PEEP trial and EIT measures. *Top*, global impedance waveform during decremental PEEP trial from 14 cmH_2_O to 0 cmH_2_O with decremental steps of 2 cmH_2_O. At each step, EIT data were analyzed. *Mid-top*, EIT tidal images corresponding to each PEEP level. Calculated EIT measures are shown with values and images. *Mid-bottom* and *bottom*, images depict the center of ventilation (CoV) and overdistension/collapse (ODCL) at each PEEP level. In the ODCL images, blue and white indicate sites affected by overdistension and collapse, respectively. The first value represents overdistension (%) and second value represents collapse (%)

The cumulated overdistension is calculated in the same way. By labeling those affected pixels showing reduced compliance at each PEEP level in the EIT image, the areas of possible ODCL are visualized (Fig. 2). In addition, quantitative evaluation becomes possible by calculating and accumulating the reduction in pixel compliance from the highest value. Thus, theoretically, the ideal PEEP level at which the minimum ODCL occurs could be identified by means of this analysis when performing a PEEP trial.

Costa and colleagues performed EIT measurement and chest computed tomography (CT) simultaneously and compared the results using this technique during a decremental PEEP trial in two ARDS patients [15]. They found the location and quantification of regional collapse and overdistension detected by EIT correlated well with that calculated from CT data. When PEEP was decreased, the pixels in the dorsal region were affected more by collapse, and increased PEEP led to increased overdistension in the ventral region of both patients. These results support the premise that EIT can detect alveolar collapse and overdistension at the bedside when changing the level of PEEP. It is of note that although there was a reasonable correlation in detection of overdistension between CT and EIT, a discrepancy still existed. The authors pointed out that CT-derived overdistension might be underestimated because the parenchymal density threshold for overdistension is given based on anatomical, static densities that assume that the occurrence of the phenomenon would follow an all or none principle. In fact, overdistension is naturally a functional measure of excessive tissue stretch. In the study by Costa et al., EIT revealed such a non-physiological abnormal overstretch of the lung parenchyma functionally in accordance with increased PEEP, which characterized EIT as a unique monitoring tool. However, it is uncertain whether EIT has possibilities to manifest the physiological limitation of overstretch beyond which lungs enter a pathophysiologic state leading to VALI.

#### Center of ventilation (CoV)

The center of ventilation (CoV), which was originally introduced by Frerichs et al., represents a vertical shift of the ventilation distribution along a gravitational axis. They created ventilation profiles using 64 ROIs equally divided between the right and left halves of functional scans, which elucidate how ventilation distribution deviates from a geometric center of the chest’s diameter by simple visual examination [16] (Fig. 2). When the location of CoV is expressed as a percent of the dorsal-to-ventral thorax diameter, a value of 50% represents equal distribution between the ventral and dorsal regions, whereas lower values indicate a shift of ventilation distribution towards the dorsal region. For instance, when dorsal atelectasis develops, the CoV moves ventrally showing a value greater than 50%, indicating less ventilation in the dorsal region.

CoV has been used in many experimental and clinical studies for evaluating recruitment or derecruitment of the lung [16–20]. Frerichs et al. examined the effect of exogenous surfactant and RM on the distribution of regional lung ventilation in ALI-induced newborn piglets [16]. They found that the combination of surfactant administration with RM significantly improved the ventral shift in ventilation distribution and asymmetry in the right-to-left lung ventilation distribution. In addition, oxygenation and respiratory system compliance in the recruitment group showed improvement but there was no improvement in the group without RM, indicating non-ventilated lung areas in the dorsal regions. This suggests that in supine subjects with injured lungs, a dorsal shift resulting in more homogeneous distribution may play a role in maintaining pulmonary gas exchange and improving respiratory system mechanics.

#### Global inhomogeneity index (GI index)

The global inhomogeneity (GI) index represents the spatial extent and dispersion in the distribution of tidal breath, i.e., the overall degree of spatial heterogeneity of ventilation. This EIT measure is computed according to the impedance variation in each pixel representing the volume of distributed air within predefined lung regions. The differences in impedance variation between each pixel and median value of all pixels are calculated and normalized to the sum of impedance values according to the formula below [21],

$$ \mathrm{GI}=\frac{\sum_{x,y\in \mathrm{lung}}\mid {\mathrm{DI}}_{x,y}-\mathrm{Median}\ \left({\mathrm{DI}}_{\mathrm{lung}}\right)\mid }{\sum_{x,y\in \mathrm{lung}}{\mathrm{DI}}_{x,y}} $$

(DI = value of the impedance variation, *x* and *y* = the DI of pixel xy, DI_lung_ = DI of all pixels in the pre-defined lung)

The GI index directly represents global inhomogeneity in tidal ventilation. Because the distribution of tidal breath in the whole lung is affected by various situations, such as severe inflammation, altered lung function, or different ventilator settings, the GI index varies depending on the physiologic state of the lungs. A smaller GI index represents a more homogeneous distribution, and a larger GI index indicates a more inhomogeneous ventilation.

The GI index, introduced by Zhao et al., has been employed in studies to evaluate the optimal PEEP selection, the effect of general anesthesia on ventilation distribution in pediatrics, and the progression of obstructive lung disease [21–24]. Zhao et al. investigated the feasibility of using the GI index to identify optimal PEEP during an incremental PEEP trial in patients with healthy lungs. PEEP was increased from 0 to 28 cmH_2_O, with an incremental increase of 2 cmH_2_O [21]. The GI index showed a parabolic curve in response to increased PEEP. The PEEP level at which the GI value was minimal corresponded to the highest global dynamic compliance, indicating that the optimal PEEP level, providing less inhomogeneity, could be detected by monitoring the GI.

### EIT measures for analyzing temporal distribution of ventilation

#### Regional ventilation delay (RVD)

When atelectatic areas exist, there is a delay in the distribution of inspired air in the lung. Such a temporal delay can be recognized in real-time EIT images at the bedside; however, specific measures that can quantify the degree of delay are necessary to evaluate ongoing ventilation more accurately. The regional ventilation delay (RVD) is an EIT measure that can show temporal delay in regions of the lung, i.e., the temporal heterogeneity occurring in the ventilated lung by focusing on the relationship between impedance change and the ventilation time course in each pixel. It monitors the delay in regional impedance to reach a certain impedance change, which is normally set at 40% of its maximal impedance value during a slow inflation maneuver [25]. RVD is calculated for each pixel using the formula [25]:

$$ {\mathrm{RVD}}_{\mathrm{i}}=\frac{{\Delta  t}_{\mathrm{i}}^{40\%}}{t_{\mathrm{max}}-{t}_{\mathrm{min}}}\times 100\% $$

(${\Delta  t}_{\mathrm{i}}^{40\%}$ = the time from the global start of inspiration to reaching a 40% regional impedance change of its maximum value, *t*_max_ − *t*_min_ = inflation time, i = each pixel)

Regional ventilation delay inhomogeneity (RVDI) is defined as the standard deviation of RVD in all pixels. A smaller RVDI indicates a more homogeneous distribution. Furthermore, some EIT machines can plot the area affected by ventilation delay in a color-coded RVD map; therefore, one can see the exact site where the delay is occurring and its degree of delay by the scaled color on the map.

RVD has been used as another EIT measure in research investigating the homogeneity of ventilation [25–27]. Muders et al. measured RVD using a slow inflation technique in mechanically ventilated pigs before and after induction of ALI [25]. Animals were ventilated at different PEEP levels, and RVD was monitored at each PEEP level. They found delays predominantly in the dorsal regions parallel with decreased PEEP; this was not found before ALI was induced. They also performed end-expiratory and end-inspiratory CT scans at each PEEP level to quantify tidal recruitment of the lung and compared it with RVDI. As a result, RVDI correlated well with the amount of tidal recruitment intra- and inter-individually. The authors of that study suggested that monitoring RVDI could be useful to estimate the amount of cyclic tidal recruitment and collapse that should be minimized in injured lungs to avoid VALI.

#### Intratidal gas distribution (ITV)

Intratidal gas distribution (ITV), originally described by Löwhagen et al., exhibits the way the inspired gas distributes in the lungs during the tidal breath at each region [28]. Being different from other EIT parameters, ITV delineates the changes in regional compliance with time course during one breath in different regions of interest. To calculate ITV, the inspiratory part of the global TIV curve is divided into eight iso-volume parts. Thereafter, the sequential corresponding time points of the eight iso-volume steps are translated to the regional TIV curves. With this analysis, the percentile contribution of the inspired air distributing to the selected lung region to a certain time point is calculated as below [28],

$$ \mathrm{Fractional}\ \mathrm{regional}\ {\mathrm{ITV}}_{1-8}=\frac{{\mathrm{ITV}}_{1-8}\ {\mathrm{TIV}}_{\mathrm{ROI}}}{{\mathrm{ITV}}_{1-8}\ {\mathrm{TIV}}_{\mathrm{Global}}} $$

**Fig. 3:**
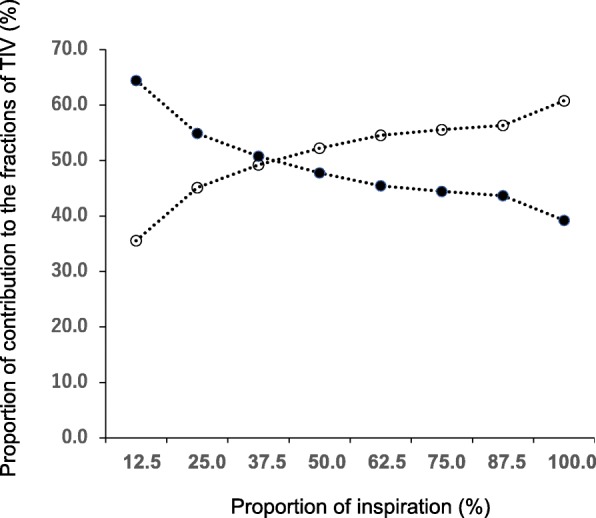
Intratidal gas distribution (ITV) curve of a patient with spontaneous breathing. The ITV curve represents the percentile contribution (%) of ventilation distribution in non-dependent and dependent lung regions during the entire inspiration. Dashed lines represent the interpolation lines; open circles = the non-dependent region; solid circles = the dependent region

When the lung region is divided into two ROIs, i.e., the ventral and dorsal regions, impedance changes from the beginning of inspiration to a certain time point in each region are expressed as a fraction of the contribution to global impedance change. For example, in spontaneous breathing with healthy lungs, the air distributes more to the dorsal region owing to the active movement of the diaphragm at the early phase of inspiration, showing more than a 50% contribution in the dorsal region to the global TIV (regional TIV < 50% in the ventral region) (Fig. 3). After being well inflated in the dorsal region somewhere in the middle of inspiration, more air then goes to ventral region and the contribution of the dorsal region decreases, showing less than a 50% contribution at the late phase of inspiration. The change in the percentile contribution of the inspired air distributing to each region during the entire inspiration can be visualized as a sequential line graph (*x*-axis, time from the start of inspiration; *y*-axis, percentile contribution to global TIV). In this case, two lines (ITV curves) representing the contribution of the ventral and dorsal regions to the inspiration are plotted on the graph and the behavior of the ITV curves is reflective of the characteristics of regional ventilation [29–32].

### Using EIT measures in clinical practice

Clinical research and case reports have demonstrated the usefulness of EIT monitoring in the respiratory management of patients with ARDS and in patients without pulmonary pathology. ARDS lungs have large regional mechanical heterogeneity; therefore, determining ventilator settings to achieve uniform ventilation distribution in ARDS is valuable to prevent VALI. Various EIT research groups have been focusing on seeking optimal ventilatory settings by using EIT measures in experimental and human studies [15, 21, 33–37]. In assessing ventilation, EIT measures not only showed comparable results with those derived from global parameters, but also can add beneficial analysis, indicating that EIT measures might have a potential to provide useful clinical information in actual clinical practice. The possible benefit in using EIT measures include the following points: (1) EIT measures can be analyzed by dedicated software once users record the impedance data, (2) trends of those calculated values can be evaluated repeatedly at any given time during the clinical course, and (3) analyzed images showing affected sites of each EIT measure (Fig. 2) promote a better understanding of the existing pathological condition and help clinicians choose a subsequent strategy of respiratory management. By referring to those profiled images and sharing the information with involved medical staff, a personalized ventilation strategy can be discussed. Though this has not been proven yet, the evaluation for ongoing ventilation by using multiple EIT measures with different characteristics might be more feasible than using a single EIT measure (Table 1). In fact, multiple EIT measures can be analyzed simultaneously, allowing users to access usable information instantly in some EIT machines. This might improve the understanding of the ongoing lung status and optimal ventilator settings. However, a prospective study is needed to confirm this.

**Table 1:** Summary of EIT measures and descriptions introduced in this review

EIT measures	Description
Tidal impedance variation (TIV)	Impedance change during a tidal breath, the difference of impedance between end-inspiration and end-expiration
Regional respiratory system compliance	Regional compliance calculated by dividing regional TIV by driving pressure
Overdistension and atelectasis/collapse (ODCL)	Affected area by overdistension or collapse representing reduced compliance by PEEP trial
Center of ventilation (CoV)	Vertical shift of the ventilation distribution along the gravitational axis
Global inhomogeneity index (GI index)	Spatial extent and dispersion in distribution of tidal breath, the overall degree of spatial heterogeneity of ventilation
Regional ventilation delay (RVD)	Temporal delay in distribution of inspired air to reach a certain impedance change
Intratidal gas distribution (ITV)	Changes in fraction of regional TIV with time course during inspiration

#### Assessment of homogeneous ventilation using PEEP trials in recruitable lungs

The most frequent approach used to determine the optimal PEEP at which inhomogeneous ventilation is minimized is the PEEP trial (Fig. 2). An EIT-guided method is based on the relative changes of lung impedance value during ventilation and is clearly different from the method of determining from a specific, absolute value like transpulmonary pressure. All the EIT measures explained above show a favorable response to PEEP trials when lungs are recruitable. Bikker and colleagues calculated regional compliance during an incremental/decremental PEEP trial and found a different response in regional compliance between dorsal and ventral lung regions, and between ARDS and non-ARDS patients, at identical PEEP levels [33]. Wolf and coworkers investigated the relationship between mean airway pressure during high-frequency oscillatory ventilation and regional ventilation in a swine ALI model by using a PEEP trial [18]. They found a shift of CoV from a nondependent to dependent area when increasing the mean airway pressure from 5 cmH_2_O to 40 cmH_2_O, in 5 cmH_2_O increments, and the shift correlated well with the decrease in shunt fraction.

A PEEP trial can also be performed in the operating room to seek the optimal PEEP level. Recently, clinical research has been reported by Nestler et al., in which RVD was used for individualized ventilatory settings in anesthetized, morbidly obese patients during laparoscopic surgery [38]. Patients were randomly divided into two groups: one group ventilated with individualized PEEP at which the minimum RVDI was detected by a decremental PEEP trial with RM, and the other group ventilated with a PEEP of 5 cmH_2_O with no RM throughout the surgery. The results showed significantly better gas exchange, higher end-expiratory lung volume, and lower driving pressure in the individualized PEEP group (average 18.5 cmH_2_O), indicating more homogeneous ventilation in this group. In addition, the GI index was significantly lower in the individualized PEEP group. Thus, individualized PEEP selection might be achieved by using RVDI or the GI index in combination with a PEEP trial.

On the basis of information previously described, RVD and GI have been chosen to quantify ventilation inhomogeneity. However, one must consider that there is a limitation when using pixel-based analysis. Because all the structures in the thorax have their own resistivity to electricity, researchers need to determine the lung field for the subsequent analysis of EIT measures by selecting a certain threshold to avoid contamination from non-lung tissues. Pixel-based analyses, such as ODCL, RVD, and GI, are calculated based on such a determined area, and their results can be affected by a predefined lung ROI [39]. In addition, even if the EIT belt is positioned at an identical level of the thorax without disconnection, a pixel at the same location in the EIT image could include information from previously unrelated pixels after RM or lung collapse, which would affect the pixel-based analysis.

#### Assessing recruited lungs by the open lung strategy and determination of lung recruitability

Although there is no established methodology or confirmed evidence that improves the outcomes of patients with ARDS, RM could play a role in minimizing ventilator-induced lung injury. However, the methods for the assessing recruited lungs without taking CT are scarce in clinical practice. Evaluating the effect of RM using EIT measures has been addressed by several EIT researchers [40–42]. Because successful recruitment should result in an increase in compliance, all the studies demonstrated increased regional compliance in the dorsal region after RM. Several studies also assessed the effect of RM in combination with increased regional compliance and end-expiratory lung impedance (EELI), which is considered to correspond to end-expiratory lung volume [43, 44].

Before performing RM, determination of recruitability is required when the lungs are suspected to be vulnerable to high inspiratory opening pressures. EIT has the potential to determine recruitability without applying excessive opening pressure by measuring the regional compliance during a PEEP trial. Camporota reported two ARDS cases that required extracorporeal membrane oxygenation (ECMO) [34]. The patients showed different responses to PEEP. In one patient, the highest regional compliance was obtained at a PEEP of 8 cmH_2_O in the ventral and 22 cmH_2_O in the dorsal region. In the other patient, the highest regional compliance was not detectable; no response to PEEP change in either the ventral or dorsal regions was observed, indicating that the lungs were nonrecruitable. Another technique to estimate lung recruitability was reported by Zhao and coworkers. They conducted research using a modified GI index and examined the relationship between the GI index and lung recruitability using a constant low-flow inflation maneuver in both ARDS and lung-healthy groups [23]. The modified GI index was calculated based on the differential EIT functional images obtained between different time points during prolonged inspiration. The researchers found that when the recruitable volume decreased gradually, the GI index decreased concurrently in both groups, showing high correlation between the two values. In addition, the GI index was significantly higher in the ARDS group than that in the lung-healthy group. These studies suggest a possible use of the GI index for estimating lung recruitability in normal or pathologic lungs, and ventilatory settings aiming at the lowest value may be reasonable to provide better ventilatory settings.

#### Assessing ventilation in different ventilator modes and body positions in rescue therapy

Rescue therapies such as prone positioning, special ventilatory modes, muscle relaxants, and ECMO are applied when low tidal volume ventilation with a high PEEP cannot maintain gas exchange in severe ARDS. The effects of these therapies on regional ventilation distribution have been examined in case reports and previous research [44–47]. Kotani et al. reported the clinical use of EIT for a severe ARDS patient who developed acute cor pulmonale [46]. Despite the effort to improve hypoxemia and respiratory acidosis with several special ventilatory modes, the patient had severe right ventricular (RV) failure. Prone positioning was proposed as a rescue therapy, and EIT monitoring was performed before and after changing the body position. The authors confirmed that regional TIV in the most dorsal part successfully increased during prone positioning, demonstrating more homogeneous ventilation leading to better gas exchange and reduced systolic pressure of RV. Shono and colleagues reported a severe ARDS case with excess respiratory efforts accompanied by uncontrollable high tidal volumes, who was given a muscle relaxant as a rescue therapy [47]. Before administration of the muscle relaxant, EIT visualized the uneven distribution that shifted to the dorsal regions abnormally. The fraction of regional TIV in the dependent region was 75% of global TIV due to the hyperactive movement of the diaphragm. EIT images dynamically changed after administration of the muscle relaxant, showing increased regional TIV in the ventral part and a return of CoV to a more central position. Thus, a simple comparison of the regional TIV and CoV before and after the therapeutic intervention can be conducted when seeking better respiratory management.

#### Evaluating ventilation in assisted modes with spontaneous breathing

Previous research investigating ventilation distribution during assisted modes with preservation of spontaneous breathing has been demonstrated [48, 49]. Over-assisted ventilation by higher pressure support levels resulted in a shift of ventilation distribution to the non-dependent part in patients with mild or moderate ARDS [49]. However, in patients with severe ARDS and an extreme respiratory effort, ventilation distribution was highly different between non-dependent and dependent regions, temporally and quantitatively [47, 50]. Air was redistributed from the non-dependent part to the dependent part at the beginning of inspiration, referred to as pendelluft [50]. Therefore, when conducting an observational study associated with ARDS, including patients with severe ARDS, one must bear in mind that the results might be affected by the degree of individual strong inspiratory efforts and upon the breaths selected for analysis and EIT parameters employed.

### Considerations in data interpretation

In EIT measurements, the position of the electrode belt determines the accuracy and validity of the analyzed data. If the belt is placed at the lower thoracic level rather than in the proper position, the diaphragm may interfere with impedance changes, leading to misinterpretation of the results [51]. Additionally, even a slight displacement from the original position caused by body movement or brief removal of the belt for clinical examination interferes with accurate data acquisition. These points must be taken into account for maintaining the credibility of subsequent data analysis. Concerning the interpretation of the analyzed data, the results should be interpreted cautiously because functional EIT employs relative values, essentially different from absolute values such as transpulmonary pressure. A single measurement may not provide adequate information for judging ongoing lung status. More accurate estimation for physiological phenomena such as lung overdistention, recruitment and derecruitment could be made possible by comparing the data before (as a reference) and after intervention (e.g., RM, changing PEEP level, changing body position). In addition, the degree of impedance variation expressed by a relative color scale in functional EIT images does not necessarily delineate the intensity of the stress to the lung in corresponding regions. In fact, a small impedance variation does not always indicate insufficient under-ventilation. It is important to consider the aspect of lung volume (estimated by EELI) and its correlation with tidal ventilation when evaluating the overall homogeneity of the lungs. Moreover, additional information from other global parameters such as compliance, dead space, and blood gas exchange helps the clinician more precisely interpret the EIT data [30].

## Conclusions

Previous studies indicate regional ventilation monitoring by EIT is feasible in the intensive care setting and it has potential to lead lung protective ventilation management. Further clinical studies are warranted to evaluate whether regional ventilation monitoring using EIT can shorten the duration of ventilation or improve mortality in patients with ARDS.

## Abbreviations

ALI: Acute lung injury
ARDS: Acute respiratory distress syndrome
CoV: Center of ventilation
CT: Computed tomography
ECMO: Extracorporeal membrane oxygenation
EELI: End-expiratory lung impedance
EIT: Electrical impedance tomography
GI: Global inhomogeneity index
ITV: Intratidal gas distribution
LTV: Lower tidal volume ventilation
MSOF: Multiple system organ failure
MV: Mechanical ventilation
ODCL: Overdistension and atelectasis/collapse
PEEP: Positive end-expiratory pressure
RM: Recruitment maneuvers
ROI: Region of interest
RVD: Regional ventilation delay
TIV: Tidal impedance variation
VALI: Ventilator-associated lung injury
